# Fecal microbiota transplantation as a therapeutic modality for recurrent *Clostridioides difficile* infection: reviewing efficacy, safety, mechanisms of action, and outcomes

**DOI:** 10.1097/MS9.0000000000003649

**Published:** 2025-07-25

**Authors:** Chukwuka Elendu, Eunice K. Omeludike, Eunice T. Aregbesola, Peace Mordi, George S. Blewusi, Afeez O. Ogidan, Nzubechukwu G. Okeke, Babajide T. Obidigbo, Abigail O. Asini, Esther S. Ubi, Paul O. Etakewen, Chinobem A. Amahalu, Rogers F. Foncham, Lovert T. Gana, Chinwe J. Onwe, Ohikhuemi F. Ojeabuo, Abiola O. Ojo, Chigozie S. Ikeaba, Nnamdi C. Opara

**Affiliations:** a Department of Medicine, Federal University Teaching Hospital, Owerri, Nigeria; b Department of Medicine, Piedmont Athens Regional Medical Centre, Athens, Georgia, United States; c Department of Medicine, University of Missouri Hospital, Columbia, Missouri, United States; d Department of Medicine, Gannan Medical University, Ganzhou, Jiangxi, China; e Department of Medicine, Johns Hopkins Bloomberg School of Public Health, Baltimore, Maryland, United States; f Department of Medicine, Olabisi Onabanjo University, Ago-Iwoye, Nigeria; g Department of Medicine, Vassar Brothers Medical Center / Nuvance Health, Poughkeepsie, New York, United States; h Department of Medicine, York and Scarborough Teaching Hospital NHS Foundation Trust, York, United Kingdom; i Department of Medicine, Babcock University Teaching Hospital, Ilishan-Remo, Nigeria; j Department of Medicine, University of Calabar, Calabar, Nigeria; k Department of Medicine, King’s Mill Hospital, Sutton-in-Ashfield, United Kingdom; l Department of Medicine, University of Nigeria Teaching Hospital, Ituku-Ozalla, Nigeria; m Department of Medicine, Concept Care Solutions, Northampton, United Kingdom; n Department of Medicine, Concept Care Solutions, Watford, United Kingdom; o Department of Medicine, Kharkov National Medical University, Kharkiv, Ukraine; p Department of Medicine, University of Lagos, Lagos, Nigeria; q Department of Medicine, Mersey and West Lancashire Teaching Hospitals NHS Trust, Birmingham, United Kingdom; r Department of Medicine, Imo State University, Owerri, Nigeria

**Keywords:** antibiotic-associated diarrhea, Clostridioides difficile, fecal microbiota transplantation, gut microbiota, recurrent infection

## Abstract

Recurrent *Clostridioides difficile* infection (rCDI) remains a significant global health challenge, characterized by high morbidity, substantial healthcare costs, and an increased risk of severe complications. *C. difficile*, a gram-positive, spore-forming bacterium, is the primary cause of healthcare-associated diarrhea. The pathogenesis of rCDI is closely tied to gut microbiota disruptions, often triggered by antibiotic use, immunosuppression, and prolonged hospital stays. While effective for initial episodes, standard antibiotic therapies paradoxically exacerbate microbiota dysbiosis, increasing the risk of recurrence. Approximately 20%–30% of patients experience a recurrence after the initial episode, with rates rising to 45%–65% in those with multiple episodes. Fecal microbiota transplantation (FMT) has arrived as a transformative therapy for rCDI, leveraging donor microbiota to restore gut homeostasis and suppress *C. difficile* colonization. Clinical trials consistently report success rates exceeding 80%, markedly surpassing outcomes with antibiotics. Innovations in delivery methods, including oral capsules, have enhanced FMT’s accessibility and patient acceptability. However, concerns surrounding safety and standardization persist. Adverse events, such as gastrointestinal discomfort and rare cases of multidrug-resistant organism transmission, underscore the need for stringent donor screening protocols. Emerging evidence reveals complex mechanisms underpinning FMT’s efficacy, including restoring microbial diversity, bile acid metabolism, and short-chain fatty acid production. Long-term benefits, such as sustained microbiota stability, and potential applications in other conditions, including inflammatory bowel disease and metabolic disorders, are promising but require further validation. Addressing challenges in donor selection, regulatory oversight, and personalized approaches will be critical to optimizing FMT as a safe and effective therapeutic strategy for rCDI.

## Introduction and background

Recurrent *Clostridioides difficile* (*C. difficile*) infection presents a significant challenge in contemporary medicine, marked by substantial morbidity, heightened healthcare costs, and significant patient distress. As the leading cause of healthcare-associated diarrhea worldwide, *C. difficile*, a gram-positive, spore-forming bacterium, thrives in disrupted gut microbiomes – often resulting from antibiotic overuse, immunosuppressive therapies, or extended hospitalizations^[1–3]^. Although initial CDI episodes are effectively treated with antibiotics like vancomycin or fidaxomicin, these treatments paradoxically exacerbate microbiota imbalances, predisposing patients to recurrence. Alarmingly, 20%–30% of patients experience recurrence after their first CDI episode, with the likelihood of additional recurrences escalating thereafter.

In recent years, fecal microbiota transplantation (FMT) has gained traction as an innovative treatment for rCDI. By introducing stool from a healthy donor into a recipient’s gastrointestinal tract, FMT aims to restore microbial diversity and functionality^[4–6]^. Studies have revealed that patients with CDI exhibit reduced gut microbial diversity, including depletion of beneficial commensal organisms within the Firmicutes and Bacteroidetes phyla. FMT replenishes these depleted microbes, suppressing *C. difficile* colonization and toxin production, ultimately breaking the recurrence cycle. The effectiveness of FMT is well-documented. Clinical trials and meta-analyses report success rates exceeding 80%, surpassing conventional antibiotic therapies. For instance, a pivotal randomized controlled trial (RCT) by van Nood *et al* demonstrated an 81% resolution rate with FMT compared to 31% with vancomycin, as summarized alongside other therapies in Table 1. Innovations in FMT delivery, such as oral capsules and enemas, have enhanced accessibility and patient compliance, offering alternatives to traditional colonoscopic administration^[7–9]^. In line with current best practices for scientific integrity and the responsible integration of digital tools in research, we acknowledge the TITAN Guidelines 2025 for transparency in the reporting of artificial intelligence, while noting that no generative AI tools were used in the preparation of our work^[10]^.

**Table 1:** Comparison with alternative therapies

Parameter	FMT	Vancomycin	Fidaxomicin	Probiotics	Bezlotoxumab	Effectiveness in rCDI	Mechanism of action	Cost	References
Mechanism	Restores gut microbiota diversity and function	Inhibits bacterial cell wall synthesis	Inhibits bacterial RNA synthesis	Promotes growth of beneficial bacteria	Neutralizes *C. difficile* toxin B	Very high (up to 90% success rates)	Direct microbiome restoration	Moderate (varies by delivery method)	^[1]^
Administration	Colonoscopy, capsules, or enemas	Oral or IV	Oral	Oral	IV infusion	Effective for preventing recurrence	Recolonizes gut; restores gut homeostasis	High	^[6]^
Adverse effects	Rare but includes infection risk	GI upset, nephrotoxicity	Minimal; occasional GI side effects	Rare (bloating, gas)	Low but risk of allergic reactions	Excellent for prevention	Target microbiome manipulation	Highly variable	^[9]^
Recurrence rates	Low (10%–20%)	High (~25%–30%)	Lower than vancomycin (~10%–15%)	Limited data on recurrence prevention	Significantly reduces recurrence rates			Cost remains sensitive	^[11]^

RNA, ribonucleic acid; IV, intravenous.

The authors’ review aims to evaluate the clinical efficacy, safety, and mechanisms of action of FMT in the management of rCDI, highlighting its role as a microbiome-restorative therapy in a condition where conventional antibiotics often fail.

## Data collection

While our review process adhered to the Preferred Reporting Items for Systematic Reviews and Meta-Analyses (PRISMA) guidelines to ensure methodological rigor and transparency, a meta-analysis was not conducted due to substantial heterogeneity across the included studies in terms of study design, FMT protocols, outcome definitions, and follow-up durations. These methodological differences precluded the pooling of meaningful data and statistical synthesis; therefore, a narrative synthesis approach was adopted. To address conflicting findings, we applied the following strategies: (i) greater interpretive weight was given to systematic reviews, meta-analyses, and large-scale RCTs; (ii) discrepancies were analyzed about sample size, patient selection criteria, FMT administration route; number of treatments, and follow-up duration; and (iii) variations in microbiota composition analysis and outcome definitions were considered as potential contributors to the differences in reported efficacy or safety outcomes.

**HIGHLIGHTS**

- Fecal microbiota transplantation (FMT) increases microbial diversity and suppresses *C. difficile* overgrowth.
- Oral capsules and colonoscopy are effective delivery routes for FMT.
- FMT modulates immune responses and strengthens gut barrier integrity.

We conducted a comprehensive literature search across PubMed, Embase, Scopus, Web of Science, and the Cochrane Library for studies published between January 2000 and March 2025. The complete search strategy and the number of articles identified per database are presented in Table 2. Google Scholar was excluded due to concerns about redundancy and the inclusion of non-peer-reviewed literature.

**Table 2:** Literature search strategy and article retrieval summary

Database	Boolean search strategy used	Date last searched	Number of articles retrieved
PubMed	(“Fecal Microbiota Transplantation” OR “FMT”) AND (“Clostridioides difficile” OR “C. difficile” OR “recurrent CDI” OR “rCDI”)	28 March 2025	42
Embase	(“fecal microbiota transplantation”/exp OR “FMT”) AND (“clostridioides difficile” OR “C. difficile” OR “rCDI” OR “recurrence”)	28 March 2025	31
Scopus	TITLE-ABS-KEY(“fecal microbiota transplantation” OR “FMT”) AND (“Clostridioides difficile” OR “recurrent CDI” OR “rCDI”)	28 March 2025	27
Web of Science	TS = (“fecal microbiota transplantation” OR “FMT”) AND (“Clostridioides difficile” OR “C. difficile” OR “rCDI”)	28 March 2025	18
Cochrane Library	(“fecal microbiota transplantation” OR “FMT”) in Title Abstract Keyword AND (“recurrent CDI” OR “C. difficile”)	28 March 2025	16
Total	–	–	134

We included peer-reviewed clinical trials, cohort studies, case-control studies, systematic reviews, and meta-analyses that examined the use of FMT in managing rCDI. Only studies published in English were included. Studies focusing on animal models, non-recurrent CDI, or those reporting insufficient outcome data were excluded.

**Figure 1.**
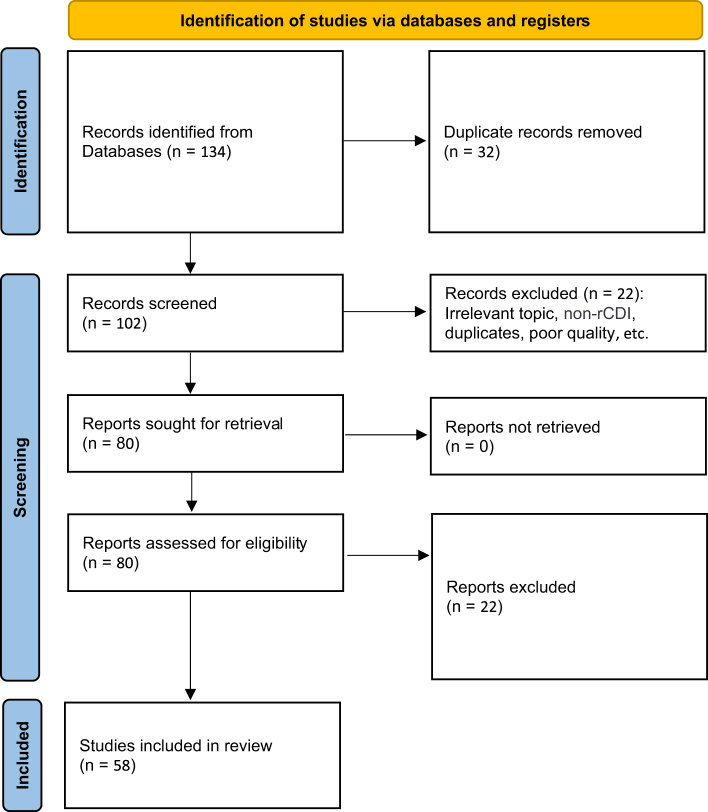
PRISMA flow diagram illustrating the process of study selection. The PRISMA flow diagram depicts the study selection process, outlining the identification, screening, eligibility assessment, and inclusion of studies considered in this review. Records were excluded for reasons including non-rCDI focus (*n* = 9), insufficient outcome data (*n* = 5), poor methodological quality (*n* = 4), previously undetected duplicates (*n* = 2), and language other than English (*n* = 2).

Two independent reviewers screened the titles and abstracts of identified studies using Rayyan for blinded screening. Full-text reviews were conducted for all articles that met the inclusion criteria. Disagreements during screening or full-text review were resolved by consensus or, when necessary, by consultation with a third reviewer. Extracted data included study design, sample size, intervention details, outcomes (efficacy and safety), and follow-up duration. Table 3 summarizes the patient selection criteria and characteristics across the included studies. The PRISMA flow diagram (Fig. 1) illustrates the study selection process, including the identification, screening, eligibility assessment, and inclusion of studies considered in this review. Records were excluded for reasons including non-rCDI focus (*n* = 9), insufficient outcome data (*n* = 5), poor methodological quality (*n* = 4), previously undetected duplicates (*n* = 2), and language other than English (*n* = 2). To ensure the validity and reliability of the included studies, RCTs were assessed using the Cochrane Risk of Bias 2.0 tool. In contrast, observational studies (cohort and case-control) were evaluated using the Newcastle–Ottawa Scale (NOS). Two reviewers independently rated each study, and discrepancies were resolved through consensus.

**Table 3:** Patient selection criteria and characteristics of included studies

Parameter	Refractory CDI	Recurrent CDI	Immunocompromised patients	Elderly patients	Pediatric patients	Pregnant patients	Severe CDI	Mild-to-moderate CDI	References
Definition	Failure of standard therapy	≥2 recurrences despite standard care	Patients with weakened immunity	Patients >65 years old	Children under 18	Pregnant or lactating women	CDI with organ failure or shock	CDI without systemic complications	^[6]^
FMT indication	Strong indication	Strong indication	Caution, assess risks versus benefits	Suitable with careful monitoring	Emerging evidence supports use	Limited data, use cautiously	Strong indication	May not be required	^[9]^
Efficacy	High	High	Effective, but immunological risks exist	Effective with fewer recurrences	Effective in severe cases	Limited evidence	High success rates	Not typically used	^[6]^
Safety considerations	Standard donor screening required	Standard donor screening required	Risk of infections or complications	Risk of comorbid conditions	Requires pediatric protocols	Lack of safety data	Safety ensured with proper protocols	Not typically applicable	^[8]^
Regulatory guidance	Recommended in guidelines	Recommended in guidelines	Requires regulatory review and consent	Supported by guidelines	Limited pediatric guidelines	Not yet standardized	Endorsed in severe cases	Not covered in some guidelines	^[12]^
Monitoring	Intensive follow-up	Regular follow-up	Close infection and complication monitoring	Routine monitoring	Requires pediatric expertise	Monitor for maternal/fetal risks	Requires critical care support	Minimal monitoring required	^[13]^
Donor selection	Strict adherence to protocols	Strict adherence to protocols	Stringent screening for pathogens	Avoid risky donors	Specialized donors	Lack of data	Carefully screened donors	Not required	^[14]^
Alternative therapies	Limited options available	Limited options available	Consider fidaxomicin or bezlotoxumab first	Antibiotics may still work	Antibiotics may be sufficient	Antibiotics preferred	May need adjunctive monoclonal antibodies	Antibiotics sufficient	^[15]^
Long-term outcomes	Promising with low recurrence rates	Promising with low recurrence rates	Unknown; needs further research	Encouraging	Promising	Unknown	Proven to reduce long-term recurrence	Not typically evaluated	^[16]^
Ethical considerations	None beyond standard	None beyond standard	Must obtain informed consent	Must balance risks and benefits	Parental consent required	Ethical concerns regarding risks	Consent from family or caregivers	Not typically relevant	^[17]^

CDI, refractory Clostridioides difficile infection; ≥ 2, greater than or equal to 2; > 65, greater than 65 years of age.

Finally, the overall quality of evidence varied. While several high-quality RCTs and meta-analyses support the efficacy and safety of FMT in rCDI, some observational studies have exhibited limitations related to sample size, follow-up duration, and reporting transparency. These limitations are further discussed in the Discussion section.

### Epidemiology of rCDI

rCDI, defined as the re-emergence of symptoms within 8 weeks of successful treatment, is a growing public health concern. Recurrence occurs in approximately 15%–35% of patients after an initial infection and increases to 45%–65% in those with multiple episodes^[1,2]^.

**Figure 2.**
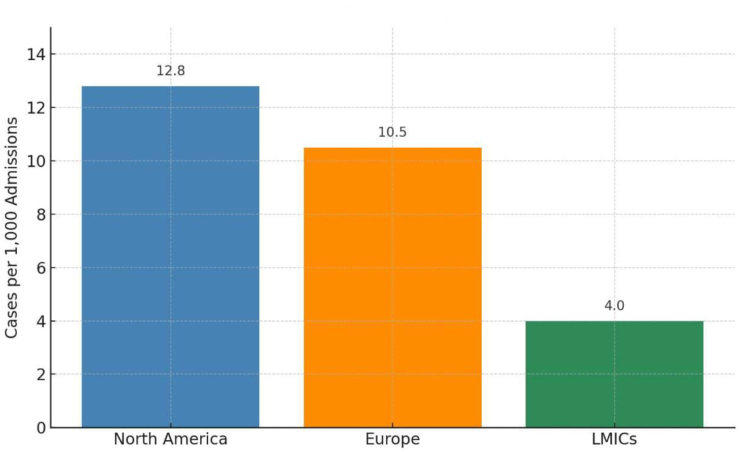
Estimated prevalence of rCDI per hospital admission by region.

Globally, the prevalence of rCDI has increased over the past two decades. In North America and Europe, *C. difficile* is among the most common healthcare-associated infections, with rates ranging from 4.5 to 12.8 cases per 1000 hospital admissions^[3,4]^. Figure 2 provides a graphical representation of the estimated rCDI prevalence per 1000 hospital admissions across North America, Europe, and low- and middle-income countries (LMICs). In the United States, there are an estimated 500 000 annual cases, resulting in over 29 000 deaths and more than $1 billion in healthcare-related costs^[5]^. About one-third of these cases are recurrent^[6]^.

In LMICs, the burden of rCDI remains underreported due to limited diagnostic capabilities. However, the increasing use of antibiotics and expansion of healthcare systems suggest a rising trend^[7]^.

Key risk factors influencing the epidemiology of rCDI include prior antibiotic exposure, advanced age, hospitalization, immunosuppression, and comorbidities such as chronic kidney disease and inflammatory bowel disease^[8–11,17–19]^. Broad-spectrum antibiotics – particularly clindamycin, fluoroquinolones, and cephalosporins – are strongly linked to disruption of gut flora and increased rCDI risk^[8,9]^. Older adults (≥65 years) account for over 60% of CDI cases and an even greater proportion of recurrences^[5,11]^. Additionally, older adults are more likely to experience severe disease manifestations and complications, including dehydration, toxic megacolon, and death, further emphasizing the importance of targeted prevention strategies in this population. Immunocompromised individuals, whether due to underlying disease or therapy, and those with frequent healthcare exposure, are also disproportionately affected^[17,18]^.

The public health implications of rCDI include higher hospital readmissions, prolonged hospital stays, and increased transmission risk within healthcare settings, especially long-term care facilities^[16,20]^. The rise of antibiotic-resistant and hypervirulent strains, such as ribotype 027, further compounds the recurrence burden and complicates infection control^[12,21]^. The global epidemiological trends underscore the need for improved surveillance, risk stratification, and targeted prevention strategies to mitigate the growing impact of rCDI^[13,14]^.

## Discussion

rCDI remains a major therapeutic challenge due to the inherent limitations of antibiotic therapy. While antibiotics such as vancomycin and fidaxomicin target *C. difficile* directly, they do not restore the underlying gut microbial imbalance that predisposes patients to recurrence. In fact, repeated antibiotic use may further disrupt the microbiota, perpetuating the cycle of relapse. FMT has arrived as a clinically rational alternative, addressing the root cause of rCDI – dysbiosis – by reintroducing a diverse and functional microbial community. This shift in treatment paradigm reflects a deeper understanding of disease pathophysiology and underscores the need to prioritize microbiome-based interventions over symptomatic antimicrobial suppression. Accordingly, the mechanisms, efficacy, and safety of FMT are examined in detail below.

### Mechanistic description

FMT is a medical procedure involving the transfer of stool from a healthy donor into the gastrointestinal tract of a recipient, aiming to restore gut microbiota balance. FMT has its roots in ancient medicine, dating back to the 4th century in China, where it was described as a treatment for severe diarrhea in the form of “yellow soup”^[14]^. Similarly, in veterinary medicine, Bedouin camel herders reportedly used feces from healthy camels to treat animals suffering from dysentery^[15]^. The modern medical application of FMT, however, began in the 1950s when Eiseman *et al*^[22]^ published the first formal case series describing the successful use of fecal enemas for pseudomembranous colitis, now known to be primarily caused by CDI. Since then, FMT has evolved into a well-established treatment for recurrent CDI, driven by a growing understanding of the human microbiome’s role in health and disease. The procedure typically involves several steps, starting with donor screening to ensure safety and minimize the risk of pathogen transmission.

Donors undergo rigorous evaluations, including medical history assessments, lifestyle screenings, and a thorough panel of laboratory tests to exclude infectious or transmissible conditions. Recommended laboratory tests include serologic screening for HIV-1/2, hepatitis A, B, and C viruses, syphilis (e.g. rapid plasma reagin or treponemal-specific tests), and *Strongyloides stercoralis*^[22]^. Stool testing is performed to rule out enteric pathogens such as *Salmonella, Shigella, Campylobacter, Escherichia coli* O157:H7, *Vibrio* species, *Yersinia enterocolitica*, and *C. difficile*. Additionally, stool samples are tested for ova and parasites, Giardia antigen, Cryptosporidium antigen, norovirus, rotavirus, and, where appropriate, SARS-CoV-2. Advanced screening may also include multiplex PCR panels and antimicrobial resistance gene profiling to reduce the risk of transmission of multidrug-resistant organisms (MDROs)^[23]^.

Once a suitable donor is identified, stool samples are collected, processed, and prepared for administration. The stool is homogenized with a saline or buffer solution, filtered to remove particulate matter, and stored as fresh, frozen, or lyophilized preparations, depending on logistical requirements^[24]^. FMT can be delivered through various routes, including colonoscopy, nasogastric or nasojejunal tubes, rectal enema, or oral capsules containing lyophilized fecal material. Colonoscopy remains the most commonly employed method, offering the advantage of direct delivery to the colon, where *C. difficile* overgrowth primarily occurs^[25]^. Oral capsule formulations, however, have gained popularity due to their noninvasive nature, ease of administration, and comparable efficacy in treating recurrent CDI^[26]^ – a summary of delivery modalities, techniques, and their clinical implications is provided in Table 4. Regardless of the route of administration, the primary goal of FMT is to restore microbial diversity and suppress *C. difficile* proliferation by re-establishing a healthy gut microbiome. rCDI is characterized by dysbiosis, a state of microbial imbalance typically involving a loss of beneficial commensal bacteria and overgrowth of pathogenic organisms, including *C. difficile*^[27,28]^. This dysbiosis is often triggered or exacerbated by antibiotic therapy, which indiscriminately reduces the diversity of bacteria in the gut. FMT counteracts this imbalance by introducing a diverse and functionally robust microbial community from a healthy donor. The transplanted microbiota replenishes depleted bacterial populations, including species within the Firmicutes and Bacteroidetes phyla, which are known to play crucial roles in gut health and homeostasis^[29]^. These commensal bacteria help to re-establish a stable ecosystem within the gut, promoting colonization resistance against pathogens such as *C. difficile*. One of the key mechanisms by which FMT exerts its therapeutic effect is the competitive exclusion of *C. difficile*. The newly introduced microbiota occupy ecological niches within the gut that might otherwise be exploited by *C. difficile*. For instance, certain commensal bacteria compete with *C. difficile* for essential nutrients, such as monosaccharides and amino acids, effectively starving the pathogen and limiting its ability to proliferate^[30,31]^.

**Table 4:** Delivery modalities and techniques

Parameter	Colonoscopy	Nasogastric/nasojejunal Tube	Enemas	Oral capsules	Efficacy	Safety	Ease of administration	Cost	References
Description	Fecal material delivered directly into colon	Fecal material introduced via gastric tubes	Rectally administered fecal material	Freeze-dried or encapsulated fecal microbiota	Very high for lower GI infections	Invasive, risk of perforation	Requires trained personnel	High (procedure and materials)	^[5]^
Indications	Recurrent or severe CDI, particularly lower GI	Suitable for upper GI involvement or severe cases	Mild-to-moderate CDI	Mild or recurrent CDI	Excellent in lower GI diseases	Can lead to aspiration in tube method	Requires setup, often uncomfortable	Varies based on region	^[6]^
Advantages	Precise delivery to target site	Non-surgical and quick	Simple, noninvasive	Noninvasive, patient-friendly	Direct administration improves outcomes	High for GI tract-wide issues	Cost	Cost-efficient by comparison	^[7]^

CDI, Clostridioides difficile infection; GI, gastrointestinal.

**Figure 3.**
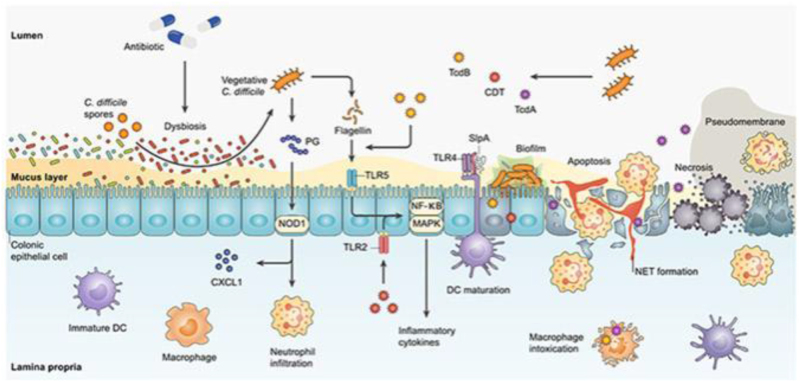
Mechanisms driving *Clostridioides difficile* (*C. difficile*) pathogenesis, including toxin production and gut microbiota disruption. This figure illustrates the main mechanisms underlying *C. difficile* pathogenesis. It highlights key processes such as toxin production (TcdA and TcdB), disruption of the gut microbiota, and inflammatory responses that contribute to disease severity. Source: Adapted with modifications from Yadegar *et al*^[1]^, reused under fair use for academic and educational purposes.

Additionally, commensals produce bacteriostatic and bactericidal substances, including short-chain fatty acids (SCFAs) such as butyrate, acetate, and propionate, which create an inhospitable environment for *C. difficile* spores and vegetative cells^[32]^. These SCFAs lower the gut’s pH, making it less conducive to *C. difficile* growth and disrupting its virulence mechanisms, such as toxin production. Furthermore, commensal bacteria can stimulate the production of antimicrobial peptides by intestinal epithelial cells, thereby enhancing the gut’s innate defense mechanisms against *C. difficile* colonization^[33]^. The main mechanisms underlying *C. difficile* pathogenesis – including toxin production (TcdA and TcdB), microbiota disruption, epithelial damage, and inflammatory responses – are illustrated in Figure 3.

Beyond microbial competition, FMT has a profound impact on the host’s immune system and gut environment. The gut microbiota plays a critical role in shaping immune responses, and dysbiosis associated with rCDI often leads to immune dysregulation^[34]^. FMT restores immune homeostasis by modulating both innate and adaptive immune pathways. For example, commensal bacteria introduced through FMT promote the production of anti-inflammatory cytokines, such as interleukin-10 (IL-10), while downregulating pro-inflammatory cytokines like tumor necrosis factor-alpha (TNF-α) and interleukin-6 (IL-6)^[35]^. This immunomodulation reduces inflammation in the gut mucosa, a hallmark of *C. difficile* infection.

Additionally, commensals enhance the integrity of the intestinal barrier by upregulating the expression of tight junction proteins, thereby preventing the translocation of pathogenic bacteria and their toxins into the systemic circulation^[36]^. The interplay between the microbiota and gut-associated lymphoid tissue is also critical. Commensals stimulate the differentiation of regulatory T cells (Tregs) and the production of secretory IgA, which contribute to the containment of *C. difficile* and the maintenance of gut homeostasis^[37]^. Emerging evidence highlights the role of microbiome-derived metabolites as mediators of FMT’s therapeutic effects. Metabolites produced by commensal bacteria can directly or indirectly influence host physiology and pathogen behavior. SCFAs, for example, serve as an energy source for colonic epithelial cells, promoting mucosal health and reducing inflammation^[38]^. They also regulate the expression of genes involved in *C. difficile* toxin production, effectively attenuating the pathogen’s virulence^[17]^. Other metabolites, such as secondary bile acids, have gained attention for suppressing *C. difficile* spore germination and vegetative growth^[39]^. Bile acid metabolism is profoundly altered in dysbiosis, with an accumulation of primary bile acids that facilitate *C. difficile* proliferation. FMT restores the balance between primary and secondary bile acids by reintroducing bile salt hydrolase-producing bacteria, which convert primary bile acids into their secondary forms. This shift in bile acid composition creates an environment that favors commensal colonization while inhibiting *the growth of C. difficile*^[40]^.

In addition to SCFAs and bile acids, other microbiota-derived metabolites, such as indole derivatives, play a role in FMT’s mechanism of action. Indoles, produced by the metabolism of dietary tryptophan by gut bacteria, have anti-inflammatory properties and contribute to maintaining intestinal barrier integrity^[41]^. They also interact with host receptors, such as the aryl hydrocarbon receptor, to regulate immune responses in the gut. Dysbiosis in rCDI is associated with a depletion of indole-producing bacteria, and FMT helps restore these populations, thereby enhancing gut health and resilience against *C. difficile*^[42]^. The expanding role of microbiome science in elucidating these mechanisms is summarized in Table 5. These interrelated changes – including increased microbial diversity, restoration of commensal bacteria (e.g. Firmicutes and Bacteroidetes), suppression of *C. difficile*, enhanced epithelial integrity, and modulation of immune responses – are illustrated in Figure 4a. Supporting data visualizations of the increase in alpha diversity and shifts in microbial composition post-FMT are shown in Figure 4b and c, respectively. Together, these mechanistic pathways illustrate the multifaceted role of FMT in restoring gut microbial ecology, enhancing mucosal immunity, and suppressing C. difficile virulence. A consolidated visual overview of these pre- and post-FMT interactions is presented in Figure 5.

**Figure 4.**
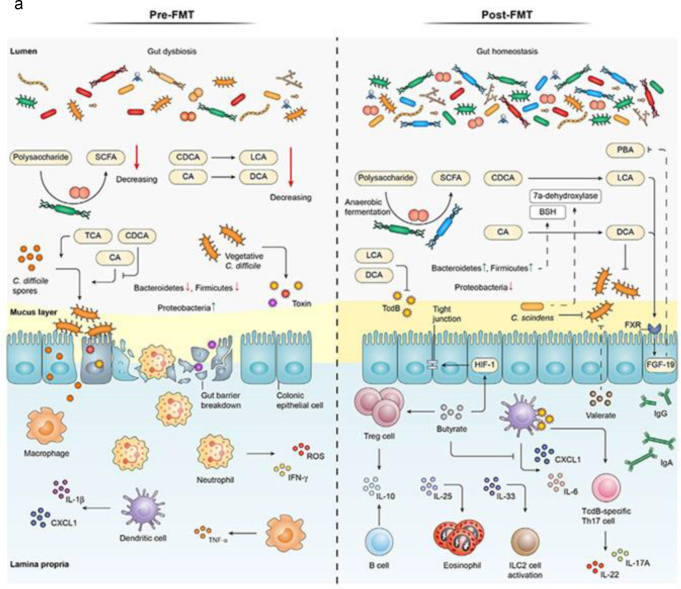

**Figure 4.**
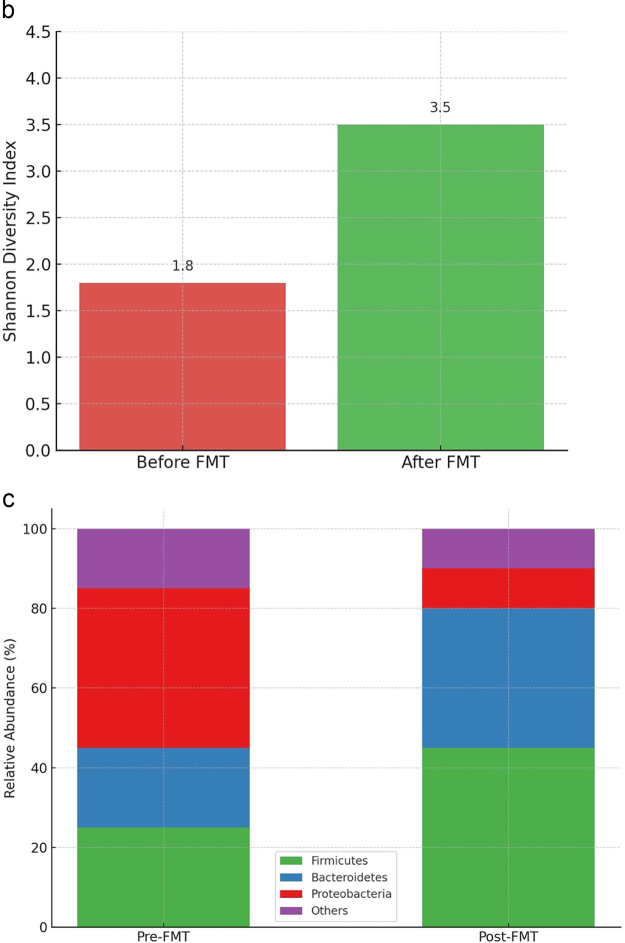
Microbiota and immune modulation before and after FMT. (a) Pre- and post-FMT interactions between *Clostridioides difficile*, the intestinal microbiota, and the immune system. (b) Microbial diversity before and after FMT based on the Shannon Diversity Index. (c) Relative abundance of key bacterial phyla before and after FMT. This figure presents the pre- and post-fecal microbiota transplantation (FMT) interactions between *Clostridioides difficile*, the intestinal microbiota, and the immune system. The left panel illustrates dysbiosis before FMT, with reduced microbial diversity, *C. difficile* overgrowth, disruption of the intestinal epithelial barrier, and heightened pro-inflammatory responses (e.g. increased TNF-α, IL-6). The right panel depicts the post-FMT state, highlighting restored microbial balance with increased Firmicutes and Bacteroidetes, re-establishment of tight junction integrity, suppression of *C. difficile* colonization, production of short-chain fatty acids (SCFAs), and immune modulation through enhanced IL-10 expression and Treg activity. Source: Adapted with modifications from Yadegar *et al*^[1]^, reused under fair use for academic and educational purposes. This bar graph shows microbial diversity (alpha diversity) before and after FMT, measured using the Shannon Diversity Index. It visually supports (a) by demonstrating the increase in gut microbiota diversity post-FMT. Source: Authors’ creations. (c) The relative abundance of key bacterial phyla before and after FMT shows an increase in Firmicutes and Bacteroidetes, a reduction in Proteobacteria minor change in other phyla. This graph supports (a) by visually reinforcing the microbiota shifts associated with FMT. Source: Authors’ creations.

**Figure 5.**
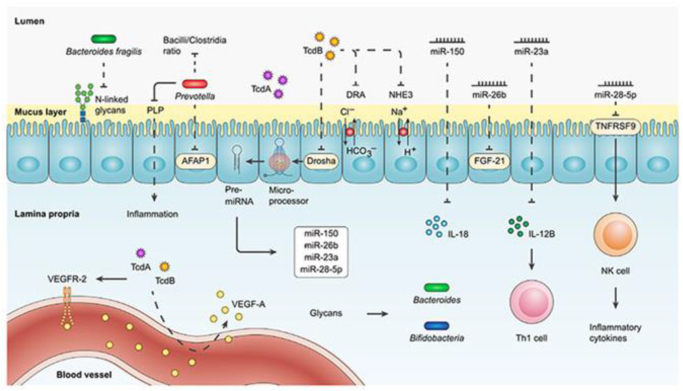
Role of miRNAs and glycans in modulating immune responses and gut microbiota post-FMT. This figure presents the pre- and post-fecal microbiota transplantation (FMT) interactions between *C. difficile*, the intestinal microbiota, and the immune system. It contrasts dysbiosis before FMT, characterized by *C. difficile* overgrowth, with post-FMT restoration of microbial balance and immune modulation. Source: Adapted with modifications from Yadegar *et al*^[1]^, reused under fair use for academic and educational purposes.

**Table 5:** Ethical and societal considerations

Parameter	Donor selection	Informed consent	Privacy concerns	Stigma around FMT	Cultural acceptance	Cost-effectiveness	Accessibility	Equity issues	Regulatory challenges	References
Description	Strict screening to avoid transmission of pathogens	Ensuring patients understand risks and benefits	Protecting donor and recipient identities	Overcoming reluctance due to fecal origin	Varies by societal beliefs and taboos	Reducing healthcare costs for rCDI	Limited availability in low-resource settings	Unequal access to FMT in underserved populations	Standardization and safety regulations	^[6]^
Ethical concerns	Risks of undetected infections or complications	Adequate disclosure of risks and alternatives	Ensuring donor anonymity	Misconceptions about the procedure	Ethical issues with cultural resistance	Justifying costs for public health	Resource allocation for FMT programs	Addressing disparities in FMT availability	Inconsistent international guidelines	^[7]^
Societal challenges	Public trust in donor screening	Patients hesitant to consent due to fear	Donor reluctance due to privacy issues	Educating the public about scientific basis	Resistance in conservative cultures	Perceived as cost-effective in long term	Limited centers offering FMT	Gaps in access between rural and urban areas	Legal and regulatory uncertainties	^[8]^
Possible solutions	Improved screening technologies	Use of standardized consent forms	Enforcing strict confidentiality policies	Public education campaigns	Cultural sensitivity inimplementation	Subsidizing FMT in public programs	Expanding infrastructure for FMT	Prioritizing equitable healthcare policies	Harmonizing regulations globally	^[9]^
Examples	Molecular screening for infectious diseases	Detailed explanation of procedures	Anonymized donor banks	Reducing stigma with patient success stories	Community engagement initiatives	Reducing recurrence saves costs	Mobile FMT units in underserved areas	Donor pools for low-resource settings	FDA and WHO initiatives	^[11]^
Impact on patients	Safer procedures	Increased trust in medical professionals	Reduced donor hesitation	Increased acceptance of treatment	Better adherence to FMT therapies	Lower costs for recurrent cases	Improved health outcomes in low-income groups	Better outcomes for marginalized patients	Uniform safety standards	^[11]^

FDA, Food and Drug Administration; FMT, fecal microbiota transplantation; rCDI, recurrent *Clostridioides difficile* infection;WHO, World Health Organization.

While FMT research primarily focuses on its effects on the gut microbiota, it is increasingly clear that its benefits extend beyond the gut. The gut-brain axis, for instance, is influenced by microbiota-derived metabolites and signaling molecules. Dysbiosis in rCDI is often accompanied by systemic inflammation and neuroinflammation, which can exacerbate symptoms such as fatigue and cognitive impairment. By restoring microbiota diversity and function, FMT may help alleviate these systemic effects; however, further research is needed to elucidate these pathways^[43]^ fully. Current guidelines from organizations such as the Infectious Diseases Society of America and the American College of Gastroenterology recommend FMT as a treatment option for patients with recurrent or refractory CDI, particularly after the failure of standard antibiotic therapies like vancomycin or fidaxomicin^[27,28]^. These recommendations are grounded in robust evidence demonstrating that FMT is highly effective, with clinical cure rates ranging from 85% to 90% in patients with recurrent CDI^[44]^. Furthermore, guidelines emphasize the importance of adhering to strict donor screening protocols and standardized preparation methods to ensure safety and minimize adverse events^[30]^.

### Efficacy

A growing body of evidence supports the efficacy of FMT for rCDI. Success rates consistently exceed 80% in numerous studies, with some RCTs reporting cure rates as high as 90% after a single FMT administration^[45,46]^. For instance, a landmark RCT conducted by van Nood *et al*^[47]^demonstrated that patients receiving FMT had an 81% resolution of diarrhea after the first infusion, compared to only 31% in those treated with vancomycin alone. After a second FMT, the cure rate rose to 94%. This trial highlighted the transformative potential of FMT and set a precedent for subsequent research. Subsequent studies have corroborated these findings. In a systematic review and meta-analysis by Cammarota *et al*, FMT achieved an overall pooled efficacy of 85% for rCDI, significantly outperforming traditional therapies like metronidazole and vancomycin^[48]^. Another meta-analysis by Quraishi *et al* reinforced these results, reporting a pooled cure rate of 92% for FMT compared to 69% for vancomycin^[49]^. These analyses underscore the robustness of FMT as a therapeutic modality across diverse patient populations and clinical settings. Comparatively, antibiotics have shown limited long-term efficacy for rCDI, primarily due to their inability to restore the balance of gut microbiota. Antibiotic therapy, while effectively eradicating *C. difficile*, fails to address the dysbiosis predisposing patients to recurrence. Recurrence rates with vancomycin and fidaxomicin remain substantial, ranging from 20% to 30% after initial treatment and increasing with subsequent episodes^[50]^.

In contrast, FMT achieves higher initial cure rates and significantly reduces the risk of recurrence. Studies have shown that patients treated with FMT experience recurrence rates as low as 5%–10%, compared to 20%–40% with antibiotics alone^[51]^. This dramatic reduction in recurrence underscores the unique ability of FMT to restore gut homeostasis and prevent reinfection. The comparative effectiveness of FMT over other therapies has also been demonstrated in studies exploring novel antibiotic regimens and adjunctive therapies. Fidaxomicin, a newer antibiotic with a narrower spectrum of activity, has shown lower recurrence rates than vancomycin; however, its efficacy still falls short of that of FMT. A study by Wilcox *et al* reported a recurrence rate of 15% with fidaxomicin, compared to 25% with vancomycin, highlighting the need for adjunctive or alternative strategies (see Table 1)^[8]^. In contrast, FMT has consistently outperformed both agents, making it the preferred treatment for rCDI in clinical guidelines^[9]^. The impact of FMT on recurrence rates is further evidenced by its success in treating patients with multiple episodes of CDI. For example, Kelly *et al*^[5]^ conducted a multicenter trial involving patients with three or more episodes of CDI. They found that FMT achieved a clinical resolution rate of 91% after one or two infusions. Importantly, the study also demonstrated sustained efficacy, with 89% of patients remaining symptom-free at 6 months.

Similarly, Hvas *et al* reported long-term remission rates exceeding 80% in 1 year, highlighting the durability of FMT as a therapeutic approach^[11]^. The efficacy of FMT has also been evaluated in specific populations, including immunocompromised patients and those with severe CDI. A detailed summary of patient-specific considerations – including efficacy, safety, regulatory guidelines, and ethical issues across pediatric, elderly, pregnant, and immunocompromised groups – is presented in Table 6. Immunocompromised individuals, including those with solid organ transplants or hematologic malignancies, are at increased risk for rCDI and often experience poorer outcomes with conventional therapies. Despite initial safety concerns, studies have shown that FMT is highly effective and well-tolerated in these populations. A systematic review by Kelly *et al* found cure rates exceeding 80% in immunocompromised patients, comparable to those observed in immunocompetent individuals^[18]^. Furthermore, FMT has shown promise in treating severe and fulminant CDI, where standard therapies often fail. A study by Fischer *et al* demonstrated a 75% resolution rate for severe CDI after FMT, compared to 50% with antibiotics alone^[17]^. These findings underscore the versatility and broad applicability of FMT in various clinical scenarios. Another notable aspect of FMT efficacy is its ability to restore gut microbiota diversity, a key factor in preventing the recurrence of CDI. Studies using high-throughput sequencing have shown that FMT restores the microbiota to a composition resembling that of healthy donors within days of the procedure^[19]^. This restoration of microbial diversity is associated with enhanced colonization resistance against *C. difficile* and improved gut barrier function.

**Table 6:** Role of microbiome research in FMT advancements

Parameter	Next-generation sequencing (NGS)	Microbiome profiling	Personalized microbiome therapies	Artificial intelligence (AI) in FMT	Donor selection	Therapy optimization	Understanding mechanisms	Predictive modeling	Global applications	References
Description	Advanced DNA sequencing techniques	Identifying microbial diversity in the gut	Tailoring treatments to individual microbiota	AI algorithms for donor-recipient matching	Ensuring safety and efficacy	Enhancing delivery methods	Decoding host-microbe interactions	Predicting FMT outcomes	Applying research to diverse populations	^[1]^
Applications	Identifying key microbial species	Understanding microbial composition in disease	Targeted FMT for specific conditions	Optimizing donor selection and matching	Reducing risks of pathogen transfer	Improved treatment success rates	Revealing key microbial functions	Reducing recurrence risks	Developing standardized FMT protocols	^[2]^
Advantages	Precision in identifying beneficial microbes	Comprehensive analysis of gut microbiota	High specificity in therapy	Faster, more accurate donor screening	Higher safety standards	Increased efficacy in rCDI treatment	Better understanding of mechanisms	Accurate patient outcome predictions	Universal application of best practices	^[3]^
Challenges	High costs and technical complexity	Requires specialized equipment	Limited clinical evidence for targeted therapies	Need for large datasets to train models	Regulatory approval complexity	Lack of standardization	Complexity of host-microbe dynamics	Data variability	Variability in regional microbiomes	^[4]^
Impact on FMT	Identifies beneficial bacterial strains	Enables personalized FMT	Tailors treatment to patient-specific needs	Improves donor-recipient matching accuracy	Safer and more effective treatments	Reduced recurrence rates	Enhanced understanding of outcomes	Greater precision in therapy	Wider applicability of FMT	^[5]^
Future directions	Expand microbial databases	Improve global accessibility to sequencing	Develop targeted microbial therapies	Expand AI applications in FMT optimization	Build global donor banks	Increase real-world studies	Explore interactions in gut-brain axis	Use machine learning for predictions	Standardize practices across regions	^[6]^

AI, artificial intelligence; DNA, deoxyribonucleic acid; FMT, fecal microbiota transplantation; rCDI, recurrent Clostridioides difficile infection.

Additionally, FMT has increased the abundance of beneficial bacteria, such as Bacteroides and Firmicutes, while reducing pathogenic taxa associated with dysbiosis^[52]^. The durability of FMT’s effects is further supported by its impact on quality of life and healthcare utilization. Recurrence of CDI is associated with significant morbidity, healthcare costs, and diminished quality of life. By achieving sustained remission, FMT reduces the need for repeated hospitalizations, antibiotic courses, and outpatient visits. A study by Mamo *et al* demonstrated a 40% reduction in healthcare costs within 1 year of FMT, highlighting its cost-effectiveness as a therapeutic intervention^[20]^.

### Safety

Reported adverse events following FMT range from mild, self-limiting symptoms to rare but serious complications. Common adverse events include abdominal discomfort, bloating, diarrhea, and flatulence, which typically resolve within a few days post-transplantation^[50]^. These mild symptoms are often attributed to the initial microbial engraftment process and are generally not a cause for concern. However, there have been reports of severe adverse outcomes, including infections and systemic inflammatory responses. Infections are particularly concerning as the donor stool may harbor undetected pathogens, even after rigorous screening. For instance, cases of MDROs, such as extended-spectrum beta-lactamase (ESBL)-producing *E. coli*, have been documented. In 2019, the FDA issued a safety alert after two immunocompromised patients developed severe infections from MDROs following FMT, with one case resulting in death^[51]^. This highlighted the critical need for stringent donor screening and stool testing protocols to minimize the risk of infectious complications. Beyond infections, there is a theoretical concern about the long-term consequences of altering the gut microbiota, including the potential for dysbiosis or unanticipated metabolic effects. Although no direct causal link has been established, some studies have suggested that changes in the microbiota following FMT may influence metabolic and immune pathways, possibly contributing to conditions such as obesity or autoimmune diseases^[53,54]^. These risks remain speculative and require further investigation through long-term follow-up studies. The cornerstone of FMT safety lies in robust donor screening protocols designed to identify and exclude individuals with potential health risks. Donor eligibility criteria typically include a comprehensive medical history, physical examination, and laboratory testing. Donors are screened for infectious pathogens, such as *C. difficile*, hepatitis A, B, and C, HIV, syphilis, enteric bacteria, viruses, and parasites^[55]^.

Emerging evidence suggests the need for expanded testing to include MDROs, especially given their global prevalence and the risk of transmission through FMT. Donors are also assessed for lifestyle factors and behaviors that may increase the risk of transmissible diseases, such as recent antibiotic use, travel to endemic regions, or high-risk sexual practices. Stool processing and storage also play a critical role in ensuring the safety of FMT. Fresh or frozen stool samples are processed in sterile environments to minimize contamination. Cryopreservation has enabled the development of stool banks, allowing the storage and distribution of screened, ready-to-use fecal material. However, using frozen samples raises additional safety concerns, as freezing may not fully eliminate certain pathogens, such as norovirus or spore-forming bacteria^[56]^. Research is ongoing to standardize stool preparation methods and optimize the preservation of beneficial microbial components while minimizing risks.

Regulatory perspectives on FMT vary across regions, reflecting differences in healthcare systems, cultural attitudes, and scientific consensus. In the United States, the FDA classifies FMT as a biologic therapy and has implemented guidelines for its use in treating rCDI. While the FDA currently exercises enforcement discretion for FMT in rCDI cases that do not respond to standard therapies, the agency mandates that stool banks and clinical providers adhere to strict screening and documentation requirements^[57]^. This regulatory framework aims to strike a balance between patient access to FMT and the need for rigorous safety oversight. In contrast, European countries have adopted a more decentralized approach to FMT regulation. FMT is generally classified as a medicinal product in the European Union, but regulatory standards differ among member states. For example, the Human Microbiome Project has advocated for establishing centralized stool banks in the United Kingdom to streamline donor screening and ensure consistency in FMT practices^[58]^. Similarly, Australia has implemented national guidelines for FMT, emphasizing the importance of donor screening, traceability, and patient monitoring.

### Discussion of aims

Numerous studies have provided robust evidence supporting the sustained efficacy of FMT in preventing recurrent episodes of rCDI over extended follow-up periods. A systematic review of 37 studies involving more than 1000 patients demonstrated that FMT achieved a long-term cure rate exceeding 90% for rCDI, with recurrence rates remaining below 10% at 12 months post-treatment^[1]^. Similar findings were reported by Fischer *et al*, who found that a single FMT infusion resulted in durable clinical resolution in over 85% of patients at 6 months, and 81% of patients maintained symptom-free status for up to 2 years^[2]^. The sustained efficacy is attributed to restoring a healthy and diverse gut microbiota, which serves as a natural defense against *C. difficile* colonization and proliferation. Patient-specific factors, including underlying comorbidities, concurrent antibiotic use, and adherence to dietary modifications, also influence the long-term success of FMT. Patients with robust immune systems tend to have better long-term outcomes than those with immunocompromised conditions. While most studies focus on short- to medium-term outcomes, long-term prospective studies remain limited. However, follow-up data from retrospective analyses suggest that even after 3 years, recurrence rates among FMT recipients remain significantly lower than those observed with antibiotic therapy alone^[3]^. FMT exerts its therapeutic effects by restoring microbial diversity and stability in the gut. The disrupted gut microbiota in rCDI is characterized by a loss of commensal bacteria, such as Bacteroides and Firmicutes, and an overgrowth of pathogenic species, including *C. difficile*. FMT introduces a diverse consortium of microorganisms that repopulate the gut and suppress pathogenic species through competitive exclusion, the production of antimicrobial compounds, and the modulation of gut metabolites.

Long-term studies indicate that the microbial composition of FMT recipients closely resembles that of the donor microbiota within weeks of the procedure, with sustained colonization observed for up to a year or more^[4]^. Analysis of stool samples from FMT recipients shows that bacterial diversity increases significantly within the first month post-transplant and stabilizes over time, with a marked reduction in *C. difficile* spore load^[5]^. This microbiota stability is crucial in maintaining gut homeostasis and preventing subsequent infections. Interestingly, certain factors may influence the stability of the gut microbiota post-FMT. For example, continued exposure to antibiotics has been shown to reduce microbiota diversity and potentially diminish the long-term benefits of FMT. Conversely, dietary patterns rich in fiber and prebiotics have been associated with enhanced microbiota stability and improved clinical outcomes^[6]^. Furthermore, recent studies utilizing advanced sequencing technologies have identified specific microbial taxa, such as *Faecalibacterium prausnitzii* and *Akkermansia muciniphila*, as key contributors to the therapeutic success of FMT. Beyond its efficacy in treating rCDI, FMT has shown promise in managing a range of other conditions linked to gut dysbiosis, including IBD, metabolic disorders, and even neuropsychiatric conditions. FMT has demonstrated modest efficacy in patients with ulcerative colitis (UC), with approximately 30% achieving clinical remission in RCTs^[7]^.

However, long-term outcomes in this population remain mixed, with some patients experiencing sustained remission while others relapse. Factors influencing these outcomes include the microbiota composition of the donor, disease severity, and host immune responses. The potential of FMT to modulate systemic metabolic pathways has garnered significant interest. In animal models, FMT from healthy donors has been shown to improve insulin sensitivity and reduce adiposity, suggesting a role in managing metabolic syndrome and type 2 diabetes^[8]^. Preliminary clinical studies have reported similar findings, with improvements in glycemic control and lipid profiles in individuals receiving FMT for metabolic disorders^[9]^. These benefits are believed to result from the restoration of gut-derived metabolites, such as SCFAs, which regulate energy metabolism and inflammatory pathways. Despite its promising applications, the long-term risks of FMT remain a concern. One potential risk is the unintended transmission of pathogens or microbial genes, particularly in cases where donor screening protocols are insufficiently rigorous. A case report documented the transmission of multidrug-resistant *E. coli* following FMT, underscoring the need for stringent safety measures^[5]^. Additionally, concerns have been raised about the potential for FMT to alter the gut-brain axis, potentially influencing neuropsychiatric conditions. While some studies suggest that FMT may alleviate symptoms of depression and anxiety, the long-term neuropsychiatric effects remain poorly understood^[11]^. Another emerging area of interest is the potential role of FMT in modulating the gut microbiota to enhance the efficacy of immunotherapies for cancer. Preliminary data suggest that FMT may enhance responses to immune checkpoint inhibitors in patients with metastatic melanoma, with ongoing studies investigating its potential application in other malignancies^[18]^. However, these findings are preliminary, and robust evidence from large-scale, long-term studies is needed before FMT can be widely adopted in oncology.

## Challenges and limitations of study

One of the primary challenges in FMT is the limited availability of donors. The procedure relies on collecting stool samples from healthy individuals; not all individuals qualify as suitable donors. Rigorous screening protocols are necessary to minimize the risk of transmitting infectious diseases or other conditions through FMT. Donors must undergo extensive testing for bacterial, viral, and parasitic pathogens, as well as for markers of chronic diseases such as IBD or IBS. This exhaustive screening process limits the pool of eligible donors and increases the cost and logistical complexity of FMT programs^[1,2]^.

Furthermore, ethical considerations arise regarding the collection and utilization of stool samples from donors. Potential donors may be hesitant to participate due to the stigma associated with providing fecal material or concerns about privacy and the misuse of their biological material. Another significant challenge is the lack of standardization in preparing and administering fecal material. FMT protocols vary widely across institutions and practitioners, with differences in donor screening methods, stool processing techniques, and routes of administration (e.g. colonoscopy, enema, naso-enteric tube, or oral capsules). The variability in these factors can lead to inconsistencies in clinical outcomes and complicate the interpretation of study results. Standardized guidelines for FMT preparation and delivery are necessary to ensure reproducibility and reliability; however, achieving consensus remains challenging due to the diversity of practices and the limited availability of high-quality evidence^[3,4]^.

Stool banks, which serve as centralized repositories for donor material, face regulatory and operational challenges. For instance, the FDA classifies stool used in FMT as a biological product, requiring stool banks to adhere to stringent manufacturing and storage standards^[5]^. These regulations, while essential for ensuring safety, can hinder the scalability and accessibility of FMT. Despite rigorous donor screening protocols, the risk of transmitting pathogens through FMT remains a significant concern. In rare cases, FMT has been associated with the transmission of MDROs, leading to severe infections and even fatalities in immunocompromised patients^[6]^. For example, a widely reported case involved two patients who received FMT containing *E. coli* resistant to ESBLs, resulting in one patient’s death^[7]^. These incidents highlight the importance of comprehensive microbial testing of donor stool and the need to develop strategies that minimize risks, such as the use of synthetic or defined microbial communities. However, current testing methods may not detect all potential pathogens, particularly novel or emerging microorganisms, leaving room for unintended consequences. Moreover, the microbiome is a complex and dynamic ecosystem, and introducing donor microbiota into a recipient may have unpredictable effects on host physiology and immune responses.

Furthermore, emerging evidence suggests that variations in donor microbiota – such as age, diet, geographic location, lifestyle, and even antibiotic exposure – can influence the therapeutic efficacy of FMT. Donors from different regions may harbor distinct microbial communities shaped by their environment and diet, which may not uniformly engraft in all recipients. Age-related differences in microbial diversity and composition may also affect the potency and resilience of transferred microbiota^[20]^. These inter-donor differences introduce variability in clinical outcomes, yet standardized criteria to define the “ideal donor” remain elusive. A more refined understanding of how these variables impact engraftment and treatment success is crucial to improving donor selection and optimizing FMT protocols^[12]^.

Another critical limitation of FMT is the lack of robust long-term safety data. While short-term outcomes following FMT for rCDI are overwhelmingly positive, with cure rates exceeding 80%–90% in many studies, the long-term consequences of altering the gut microbiota remain poorly understood^[8,9]^. Changes in the gut microbiome induced by FMT may persist for years, potentially influencing the risk of developing other conditions such as metabolic disorders, autoimmune diseases, or malignancies. For instance, some studies have raised concerns about the potential for FMT to induce dysbiosis or disrupt host-microbiota homeostasis in ways that promote the growth of pathogenic or pro-inflammatory bacteria^[5]^. Additionally, the long-term effects of FMT on the recipient’s immune system are unclear, particularly in individuals with underlying conditions or genetic predispositions that may amplify the risk of adverse outcomes. The lack of long-term data also extends to the durability of FMT’s therapeutic benefits. While many patients experience sustained remission from rCDI after FMT, others may experience recurrence months or even years later, necessitating additional treatments^[11]^. This variability underscores the need for longitudinal studies to evaluate the stability of microbiota changes and the factors that influence long-term outcomes. Moreover, the potential for horizontal gene transfer between donor and recipient microbiota raises additional safety concerns, as it could lead to the spread of antibiotic resistance genes or other undesirable traits^[18]^.

Moreover, environmental and hospital-based factors play a substantial role in rCDI recurrence. Suboptimal infection control practices, inadequate environmental cleaning protocols, and poor air filtration can facilitate the persistence and transmission of *C. difficile* spores within healthcare settings^[22]^. Evidence suggests that interventions such as the use of sporicidal disinfectants, ultraviolet (UV) light for terminal room disinfection, and high-efficiency particulate air filtration can significantly reduce the risk of reinfection. The implementation of strict hand hygiene protocols, patient isolation strategies, and cohorting infected individuals is also associated with decreased recurrence and hospital readmissions. However, these strategies are inconsistently applied across institutions, representing a major limitation in reducing transmission and improving FMT outcomes^[23]^.

Ethical concerns also play a role in limiting the widespread adoption of FMT. Using human-derived material raises questions about donor consent, compensation, and the equitable distribution of benefits. For example, should donors receive financial compensation for their contributions, and how can this be structured to avoid exploitation or coercion? Additionally, concerns have been raised about the commercialization of FMT, with some companies developing proprietary microbiota-based therapies that may limit accessibility for patients who cannot afford them^[17]^, as summarized along with other ethical and societal considerations in Table 7.

**Table 7:** Special considerations in pediatrics and vulnerable populations

Parameter	Children with rCDI	Pregnant women	Elderly patients	Immunocompromised patients	Patients with severe CDI	Patients with co-morbidities	Donor selection challenges	Ethical considerations	Monitoring and follow-up	References
Indication	Recommended for recurrent or refractory CDI	Limited data, cautious use	High recurrence rates justify use	Risk of infections necessitates careful use	Often the best option for reducing recurrence	Requires tailored approaches	Stringent screening for pathogens	Informed consent and risk disclosure	Requires close monitoring due to risks	^[8]^
Efficacy	Promising results with low recurrence rates	Unknown; requires further studies	Effective in most cases	Effective but data on long-term outcomes are limited	High success rates in treating severe cases	Effective with comorbidity management	Limited data on optimal donor matching	Address parental or family concerns	Regular follow-up reduces complications	^[11]^
Safetyconsiderations	Ensure pediatric-specific protocols	Maternal/fetal safety concerns	Risk of adverse events due to frailty	Risk of opportunistic infections	Requires multidisciplinary care	May exacerbate certain conditions	Pathogen screening must be exhaustive	Balancing risks and benefits	Monitoring for infections or complications	^[18]^
Regulatory guidelines	Pediatric guidelines are emerging	No standardizedrecommendations	Supported by guidelines with risk assessment	Regulatory caution due to infection risks	FMT is endorsed for severe/recurrent cases	Use within approved guidelines	Stricter donor criteria required	Need for specialized protocols	Multidisciplinary team for patient follow-up	^[17]^
Ethical concerns	Parental consent required	Risks versus benefits for mother and baby	Informed consent essential	High risk requires patient/family approval	Family/caregiver approval for critical cases	Decision-making for complex conditions	Ethical concerns over donor safety	Protection of vulnerable groups	Balancing experimental and clinical use	^[19]^
Challenges	Lack of data in pediatric populations	Limited studies on pregnant patients	Comorbidities and frailty complicate outcomes	Limited evidence in immunosuppressed groups	Access to FMT in emergency settings	Multisystem involvement complicates care	Matching donors for high-risk populations	Addressing stigma and misinformation	Managing adverse events	^[52]^
Advantages	Lower recurrence and reduced antibiotic use	Potential to reduce recurrence without drugs	Effective in reducing mortality and recurrence	Reduces reliance on antibiotics	Improves outcomes in critical patients	May reduce complications of underlying disease	Improves patient outcomes significantly	Increases access to effective treatment	Ensures long-term effectiveness	^[20]^
Future directions	More pediatric-focused clinical trials	Longitudinal studies for safety and efficacy	Optimized protocols for elderly populations	Tailored approaches for immune-compromised	Expanding use in emergency cases	Research into personalized FMT	Improve donor screening technology	Address gaps in vulnerable populations	Establish global follow-up protocols	^[16]^

CDI, Clostridioides difficile infection; FMT, fecal microbiota transplantation.

Moreover, as the field evolves toward the use of synthetic microbiota and personalized donor-recipient matching, new ethical challenges arise. Informed consent becomes increasingly complex when patients are exposed to engineered microbial products or stratified donor matches based on microbiome profiling. Privacy concerns are also heightened due to the genomic data involved in personalizing FMT. Additionally, disparities in access may widen if synthetic or matched microbiota therapies are costly or limited to specialized centers, raising concerns about health equity and justice in access to cutting-edge microbiome-based treatments. Addressing these issues proactively will be critical to ensuring ethically sound and inclusive advancement in FMT technologies.

Logistical barriers further complicate the integration of FMT into routine clinical practice. The infrastructure required to collect, process, store, and deliver stool material can be resource-intensive, particularly in LMICs with strained healthcare systems. Additionally, the need for specialized training and expertise to perform FMT safely and effectively may limit its availability to select centers, creating disparities in access to care^[19]^. Efforts to develop simplified and scalable FMT protocols, such as oral capsules containing freeze-dried microbiota, are promising but remain in the early stages of development. The regulatory landscape surrounding FMT also presents challenges. In many countries, FMT is classified as an investigational therapy, requiring patients to enroll in clinical trials to access treatment. This classification limits the availability of FMT outside of research settings and may delay its adoption as a standard of care. Furthermore, the lack of consensus among regulatory bodies regarding the classification and oversight of FMT complicates efforts to harmonize practices across regions^[52]^. For instance, while the FDA regulates FMT as a biological product, the European Medicines Agency (EMA) treats it as a medicinal product, leading to differences in approval processes and requirements (see Table 8 for a summary of key regulatory and legal considerations).

**Table 8:** Regulatory and legal aspects

Parameter	FDA regulations	EU regulations	Global standardization	Donor screening	Sample processing	Patient consent	Legal liability	Ethical concerns	Future directions	References
Description	FMT classified as a drug or biological product by FDA	Governed by EMA regulations for clinical use	Lack of unified global regulatory frameworks	Rigorous screening for pathogens	Ensuring sterility and compliance with GMP	Informed consent addressing risks and benefits	Legal accountability for adverse outcomes	Balancing innovation with patient safety	Developing international consensus	^[52]^
Current guidelines	IND required unless for recurrent CDI cases	Requires approval for clinical applications	Variability in guidelines across countries	Guidelines exist but vary regionally	Standards for sample handling vary	Explicit documentation of patient approval	Providers accountable for mishaps	Ethical concerns in experimental treatments	WHO involvement in global policies	^[20]^
Challenges	Complexity of IND applications	Variation in approval processes	Inconsistent quality and safety standards	Risk of undetected infections	Contamination risks in processing	Communication barriers for patient understanding	Liability in case of donor-transmitted diseases	Potential exploitation of vulnerable populations	Harmonizing practices globally	^[16]^
Legal issues	Classification as a drug versus procedure	Divergent definitions across regulatory bodies	Patentability of FMT innovations	Privacy and confidentiality of donors	Legal liability for contamination	Ensuring legal validity of consent forms	Increased lawsuits in adverse cases	Equity in access and affordability	Addressing disparities in enforcement	^[21]^
Advantages of regulation	Enhances safety and efficacy	Improves patient outcomes through standardization	Global consistency ensures reliability	Reduces risks of infections	Prevents mishandling and contamination	Protects patient autonomy	Clarifies roles and responsibilities	Promotes trust in FMT as a therapy	Strengthens credibility of FMT globally	^[12]^
Ethicalconsiderations	Balancing innovation with patient safety	Protecting patients from harm	Equity in access across socioeconomic groups	Ensuring donor anonymity and safety	Ethical handling of biological material	Transparency in risk-benefit analysis	Balancing provider risks and responsibilities	Avoiding exploitation of marginalized groups	Encouraging responsible innovation	^[13]^
Global challenges	Lack of harmonized FDA-equivalent regulations	Disparities in access to FMT	Varying levels of infrastructure for FMT	Limited access to certified donors	Inconsistent GMP adherence globally	Inadequate communication in developing regions	Regional disparities in legal protections	Challenges in equitable access	Establishing universally recognized protocols	^[14]^
Future directions	Streamlined FDA approval pathways	Unified EU and global regulatory approaches	Creation of international regulatory bodies	Blockchain-based donor databases	Standardized GMP for global compliance	Global education on patient rights	Clear frameworks for legal accountability	Equity-focused regulatory reforms	International FMT registries for transparency	^[15]^

CDI, *Clostridioides difficile* infection; EMA, European Medicines Agency; EU, European Union; FDA, Food and Drug Administration; FMT, *fecal microbiota transplantation*; GMP, good manufacturing practice; IND, investigational new drug; WHO, World Health Organization;.

Additionally, a key limitation of our study is that it is a narrative review and not a systematic review or meta-analysis. As such, it may be subject to selection bias, and the strength of the conclusions drawn depends on the quality of the included literature. Furthermore, emerging data from studies conducted after the literature search cutoff may not be reflected in this review.

### Future research directions

One of the most notable developments in this field is the creation of standardized microbiota-based therapies, including encapsulated and synthetic microbiota. These advancements address logistical and safety challenges associated with traditional FMT, such as the need for fresh donor stool and the risk of pathogen transmission. Capsules containing lyophilized fecal microbiota have gained traction due to their ease of administration and reduced invasiveness compared to colonoscopic or enema-based methods. Studies have demonstrated comparable efficacy between encapsulated FMT and traditional routes in achieving rCDI remission^[1,2]^. Additionally, synthetic microbiota, which consists of defined bacterial consortia that replicate the therapeutic effects of FMT, is under investigation. These formulations offer the advantage of greater standardization, scalability, and reduced reliance on donor material, potentially minimizing variability in clinical outcomes^[3]^. Another promising development area is the refinement of donor microbiome profiling and selection (see Table 2).

Current donor screening protocols focus primarily on excluding pathogens and transmissible diseases; however, recent studies suggest that the composition of the donor microbiome plays a crucial role in the success of FMT^[4]^. High microbial diversity and the presence of specific bacterial taxa, such as Bacteroides and Firmicutes, have been associated with better clinical outcomes in rCDI patients^[5]^. Advances in metagenomic sequencing and bioinformatics have enabled more precise characterization of donor microbiota, allowing for the identification of “super donors” whose microbiota are particularly effective in treating rCDI. Future efforts may involve the development of predictive algorithms to match donor microbiomes with recipient profiles, thereby optimizing treatment efficacy^[6]^.

Beyond rCDI, there is growing interest in exploring the therapeutic potential of FMT and microbiota-based therapies for other diseases. Preclinical and clinical studies have suggested that the gut microbiome plays a pivotal role in various conditions, including IBD, metabolic disorders, neuropsychiatric diseases, and even cancer^[7,8]^. Emerging pilot trials have demonstrated that FMT can induce remission in patients with mild to moderate UC, with response rates ranging from 24% to 36% after multiple infusions, although results in Crohn’s disease remain more variable and less consistent^[9]^. In irritable bowel syndrome, small randomized trials have shown that FMT may reduce abdominal pain and bloating in some patients, although placebo responses and donor variability complicate the interpretation of outcomes^[16,21]^. Additional early-phase investigations are exploring FMT’s impact on hepatic encephalopathy, primary sclerosing cholangitis, and even graft-versus-host disease, underscoring the broad interest in microbiota manipulation for gastrointestinal and systemic illnesses^[12,13]^.

The modulation of gut microbiota has also been linked to improvements in insulin sensitivity and glycemic control in patients with type 2 diabetes, highlighting its potential application in metabolic syndrome^[5]^. Furthermore, the gut-brain axis has emerged as a critical area of research, with evidence suggesting that microbiota-based interventions could influence mental health outcomes in conditions such as depression and autism spectrum disorders^[11]^.

Despite these promising developments, several research gaps and challenges must be addressed to realize the full potential of microbiota-based therapies. One major limitation is the lack of long-term safety data, particularly regarding the risk of horizontal gene transfer and the emergence of antimicrobial resistance. Additionally, the mechanisms underlying the therapeutic effects of FMT are not fully understood, complicating efforts to optimize its application across different patient populations. Standardizing FMT protocols, including donor screening, preparation, and administration, remains a critical priority to ensure reproducibility and safety in clinical practice^[17,18]^. Regulatory challenges also hinder the widespread adoption of FMT and related therapies. In many countries, FMT is classified as a biological product or investigational drug, requiring extensive regulatory oversight. Streamlined guidelines that balance safety with accessibility are needed to facilitate the integration of microbiota-based therapies into routine clinical care^[19]^.

**Figure 6.**
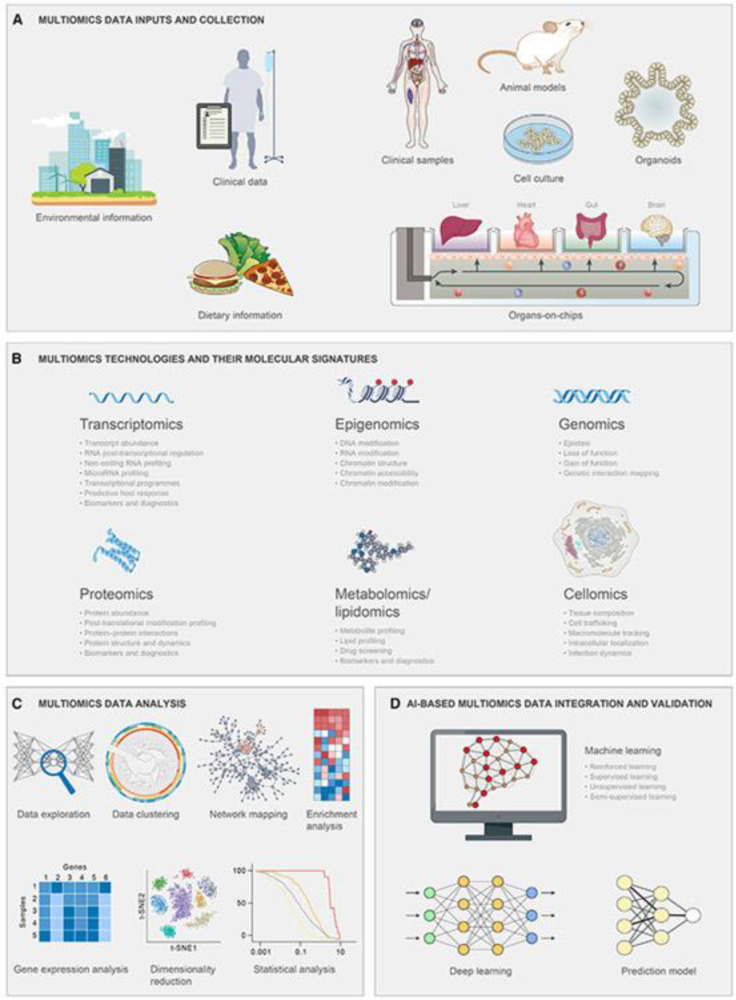
This figure demonstrates how integrated multi-omics approaches, including metagenomics, metabolomics, and transcriptomics, enhance FMT effectiveness. It emphasizes the role of data-driven strategies in optimizing patient outcomes and understanding microbiota-host interactions. Source: Created by the authors based on synthesized findings from multiple included studies^[14,17,32,34]^.

Furthermore, ethical considerations surrounding donor recruitment, informed consent, and equitable access to treatment must be carefully navigated. Equally important is addressing the limited awareness among the public and healthcare providers regarding FMT’s availability, safety, and indications – a summary of these gaps and their implications is presented in Table 9. Future research should focus on elucidating the specific microbial and metabolic signatures associated with successful FMT outcomes. Integrating multi-omics approaches (see Fig. 6), such as metagenomics, transcriptomics, and metabolomics, could provide deeper insights into host-microbe interactions and the pathways involved in disease modulation. Figure 6 illustrates how such data-driven strategies can optimize patient outcomes, improve mechanistic understanding, and support the personalization of FMT. Additionally, RCTs with larger sample sizes and diverse patient populations are needed to establish the efficacy of FMT in emerging indications. Collaborative efforts between academia, industry, and regulatory bodies will be essential to accelerate innovation and overcome existing barriers^[20,52]^.

**Table 9:** Public and healthcare provider awareness

Parameter	Misconceptions about FMT	Public perception	Healthcare provider knowledge	Patient education	Clinician training	Role of media	Community engagement	Government initiatives	Future strategies	References
Description	Misunderstandings about safety, efficacy	Varied views shaped by stigma and limited knowledge	Awareness gaps among clinicians	Providing patients with accurate and clear information	Educating clinicians about FMT practices	Media can either educate or spread misinformation	Engaging local communities for awareness	Government policies promoting FMT awareness	Strategies to address stigma and misinformation	^[14]^
Challenges	Concerns about using stool as a treatment	Cultural and societal stigma around FMT	Limited FMT education in medical curricula	Overcoming patient fears or hesitations	Limited access to formal training	Risk of sensationalized or misleading coverage	Limited outreach in rural or underserved areas	Lack of funding for awareness programs	Developing cohesive, global education campaigns	^[15]^
Educational approaches	Address safety and efficacy in public forums	Normalize FMT as a medical treatment	Provide evidence-based guidelines	Create patient-friendly resources	Incorporate FMT topics into medical education	Encourage accurate and balanced media reporting	Partner with NGOs for outreach	Support public health campaigns	Use digital platforms for widespread education	^[22]^
Impact on acceptance	Reduces fears and improves understanding	Encourages acceptance of FMT as a legitimate therapy	Enhances clinician confidence in recommending FMT	Improves patient compliance with treatments	Leads to higher adoption rates	Shapes public opinion positively	Builds trust within local populations	Increases accessibility to FMT	Promotes equity in access and use	^[23]^
Role of professional bodies	Disseminate accurate information	Partner with media and policymakers	Publish guidelines and host conferences	Empower patients with evidence-based information	Facilitate workshops and seminars	Collaborate to produce educational content	Support grassroots movements	Influence policy through advocacy	Establish global FMT education standards	^[24]^
Advantages of awareness	Promotes informed decision-making	Reduces stigma and fosters open discussions	Improves quality of care	Enables shared decision-making	Encourages appropriate clinical application	Enhances public trust in healthcare	Increases FMT access in underserved areas	Drives funding for research and implementation	Leads to better treatment outcomes	^[25]^
Ethical considerations	Ensure truthful and transparent messaging	Avoid cultural insensitivity in awareness campaigns	Prevent misinformation in clinical settings	Respect patient autonomy and decisions	Ensure inclusive training programs	Monitor for ethical reporting in the media	Avoid exploitation of vulnerable groups	Promote equitable access	Align education with ethical guidelines	^[26]^
Global opportunities	Addressing misconceptions worldwide	Increasing cross-cultural awareness	International clinician training programs	Develop multilingual patient resources	Foster collaboration across nations	Use media to highlight success stories	Promote global awareness campaigns	Support international initiatives	Innovate with technology (e.g., virtual workshops)	^[27]^
Future directions	Campaigns targeting specific misconceptions	Publicly funded media programs on FMT	FMT-specific modules in medical schools	Digital tools for patient education	Regular FMT training certifications	Partnerships with social media platforms	Grassroots advocacy for FMT	Government-backed educational grants	Integration of FMT education into global health policies	^[28]^

FMT, fecal microbiota transplantation; NGOs, non-governmental organizations.

## Final remarks

FMT is a highly effective and promising therapeutic modality for rCDI, demonstrating superior efficacy to conventional antibiotic therapies. FMT reduces recurrence rates and improves patient outcomes by restoring gut microbiota diversity and modulating host immune responses. Despite its success, challenges remain regarding standardization, safety, long-term outcomes, and regulatory oversight. Ongoing research into microbiome-based therapies, synthetic stool substitutes, and personalized treatment approaches holds promise for refining FMT and expanding its applications beyond rCDI to other microbiome-associated diseases. Optimizing patient selection, enhancing donor screening protocols, and addressing ethical, logistical, and legal considerations will be critical for the broader implementation of FMT. As the field evolves, integrating microbiome science with innovative therapeutic strategies will be essential in unlocking the full potential of FMT in clinical practice.

## Data Availability

This published article and its supplementary information files include all data generated or analyzed during this study.
